# Kawasaki Disease with an Initial Manifestation Mimicking Bacterial Inguinal Cellulitis

**DOI:** 10.1155/2020/8889827

**Published:** 2020-10-28

**Authors:** Tsukasa Tanaka, Masaki Shimizu, Oshi Tokuda, Hiroko Yamamoto, Natsuki Matsunoshita, Kanae Takenaka, Keiichiro Kawasaki

**Affiliations:** ^1^Department of Pediatrics, Kita-Harima Medical Center, Ono, Hyogo, Japan; ^2^Department of Pediatrics, School of Medicine, Institute of Medical, Pharmaceutical, Health Sciences, Kanazawa University, Kanazawa, Ishikawa, Japan

## Abstract

**Background:**

Kawasaki disease (KD) is typically characterized by fever, oral cavity erythematous changes, bilateral bulbar conjunctival injection, skin rash, erythema and edema of the hands and feet, and cervical lymphadenopathy. Some atypical patients with KD initially develop cervical and pharyngeal cellulitis; however, an initial presentation with inguinal cellulitis is extremely rare. In addition, to our knowledge, no report has documented the cytokine profile in a KD patient with cellulitis. *Case presentation*. A previously healthy 8-year-old Japanese girl was hospitalized following a 2-day history of fever and a 5-day history of pain and erythema in the left inguinal region. She was diagnosed with bacterial inguinal cellulitis and was administered antibiotics. The next day, a polymorphous rash emerged on her trunk. After 3 days of antibiotics, however, her fever continued and the cellulitis had spread over the entire lower abdomen. Simultaneously, the bilateral bulbar conjunctival injection without exudate became more prominent and her lips became erythematous. In addition, erythematous changes on her palms appeared a few hours later, which led to the diagnosis of KD. Since she had a high risk score that predicted no response to initial intravenous immunoglobulin (IVIG) at the initiation of treatment, she was treated with IVIG, intravenous prednisolone (PSL), and oral aspirin. The KD symptoms improved the next day, but the cellulitis did not completely resolve until 2 months after discharge. The patient's serum cytokine profile at admission had an IL-6 dominant pattern which was consistent with that of patients with KD despite her initial lack of KD symptoms, and the pattern observed at admission was sustained until IVIG and PSL administration.

**Conclusion:**

KD should be included in the differential diagnosis for patients presenting with inguinal cellulitis who are unresponsive to initial empiric antibiotics.

## 1. Introduction

Kawasaki disease (KD) is a systemic vasculitis with signs including fever, oral cavity erythematous changes, bilateral bulbar conjunctival injection, skin rash, erythema and edema of the hands and feet, and cervical lymphadenopathy [1, 2]. This acute febrile illness may be accompanied by some atypical inflammatory signs such as cellulitis. However, most reports of KD with cellulitis include cervical and pharyngeal cellulitis [3–6], and few reports describe KD with inguinal cellulitis [7]. In addition, to our knowledge, no report has documented the cytokine profile of a KD patient with cellulitis. Here, we describe a case of an 8-year-old girl with KD mimicking bacterial inguinal cellulitis as an initial symptom and show the cytokine profiles of this case.

## 2. Case Presentation

**Figure 1:**
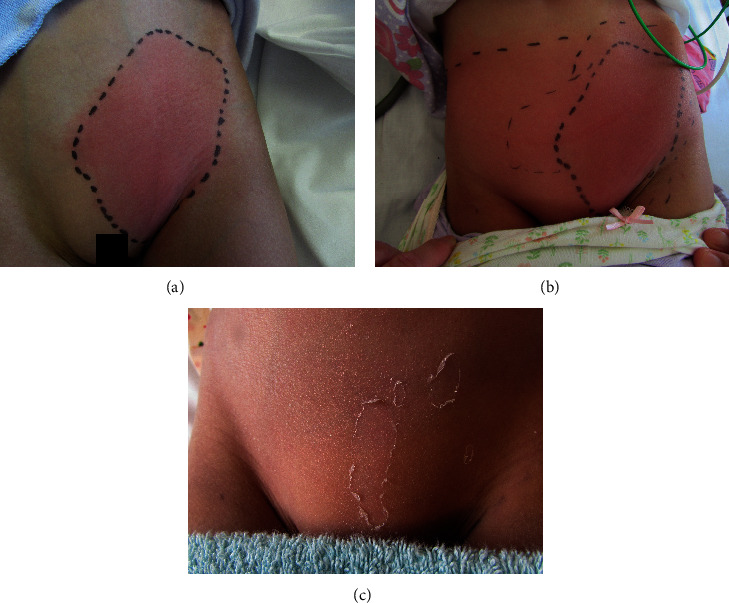
Inguinal lymphadenitis with cellulitis. (a) At admission., skin erythema was observed in the left inguinal area. (b) Before intravenous immunoglobulin treatment, inguinal cellulitis expanded to the entire lower abdomen. (c) After treatment, lamellar desquamation of the left inguinal region was observed.

A previously healthy 8-year-old Japanese girl was referred to our hospital with a 2-day history of fever and a 5-day history of pain and erythema in the left inguinal region. Physical examination on admission revealed left inguinal lymphadenitis with adjacent cellulitis (Figure 1(a)). Her skin displayed no scars from insect bites or trauma. She exhibited slight pharyngeal injection, but a rapid streptococcus test was negative. Breath sounds were clear without crackles, and no cardiac murmur was audible. A chest radiograph was unremarkable with a normal appearance of the mediastinum and no infiltrates. Initial laboratory findings were as follows: hemoglobin (Hb) 13.7 g/dL, white blood cell count (WBC) 16.0 × 10^9^/L, platelets (PLT) 264 × 10^9^/L, C-reactive protein (CRP) 11.01 mg/dL, sodium 129.5 mEq/L, aspartate aminotransferase (AST) 55 IU/L, and alanine aminotransferase (ALT) 32 IU/L.

**Figure 2:**
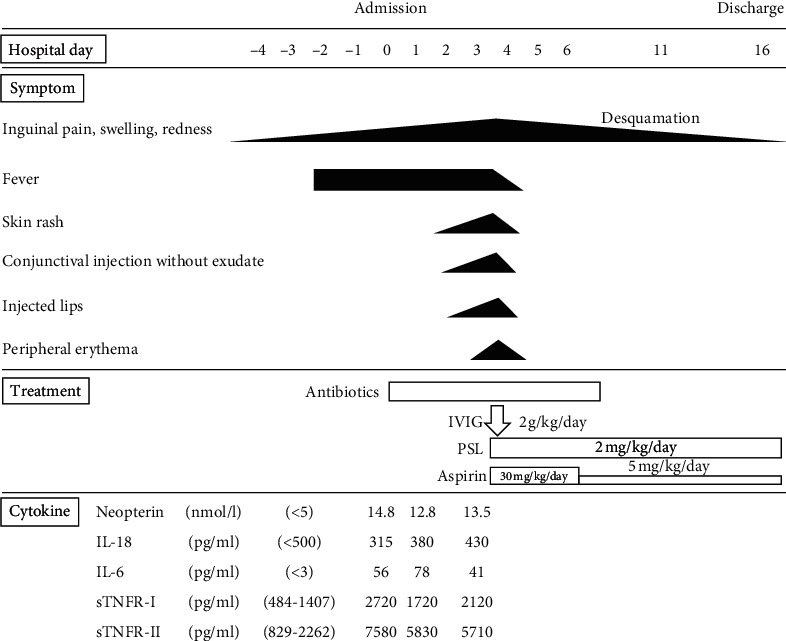
Clinical course. IL, interleukin; IVIG, intravenous immunoglobulin; PSL, prednisolone; sTNFR, soluble tumor necrosis factor receptor.

**Figure 3:**
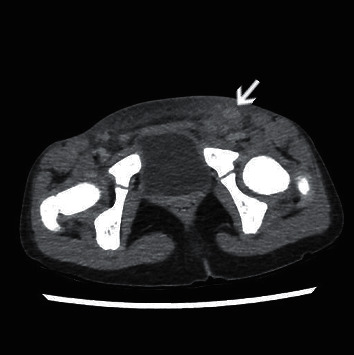
Enhanced computed tomography findings. A pelvic enhanced computed tomography scan showing an enlarged left inguinal lymph node (white arrow, approximately 1.5 × 1.0 cm), densification, and thickening of the regional soft tissue without abscess.

Her clinical course is shown in Figure 2. Physical examination findings and laboratory data seemed consistent with the diagnosis of bacterial inguinal cellulitis; therefore, she was started on intravenous ampicillin/sulbactam at a dose of 900 mg three times daily. The next day, clindamycin was administered because the cellulitis was more erythematous, and the fever persisted. Two days after hospital admission, a polymorphous rash appeared on her trunk. Antibiotics were changed to meropenem (750 mg three times daily) because of the possibility of antibiotic-resistant bacteria. Two days later, the treatment was changed to meropenem; however, she remained pyrexial. Moreover, the bilateral bulbar conjunctival injection became more prominent and her lips became erythematous. Antigen testing for adenovirus was not performed because the conjunctivital injection had no exudate. In addition, erythematous changes on her palms appeared a few hours later, which fulfilled the diagnostic criteria for KD [8]. However, the cellulitis had spread over the entire lower abdomen, which seemed atypical for KD (Figure 1(b)). Enhanced computed tomography (CT) showed no abscess formation and no necrotizing tissue. However, the increased number and size of lymph nodes and densification and thickening of the subcutaneous soft tissue in the left inguinal region on an enhanced CT scan were strongly suggestive of cellulitis (Figure 3).

In this clinical course, there was no evident septic focus other than the cellulitis. Blood cultures on admission were negative, and an enhanced CT scan showed no abscess formation. Her fever persisted despite the administration of multiple antibiotics for 3 days. Therefore, we concluded that this cellulitis was not caused by a bacterial infection. The other symptoms, such as fever lasting for more than 5 days, bilateral bulbar conjunctival injection without exudate, injected lips, a rash on her trunk, and erythematous changes on her palms, indicated that her symptoms were caused by KD, and that the inguinal cellulitis resistant to antibiotics was also caused by KD inflammation. Laboratory results at this time were as follows: WBC 12.2 × 10^9^/L (81% neutrophils, 11% lymphocytes, 5% monocytes, 2% eosinophils, and 1% atypical lymphocytes), PLT 324 × 10^9^/L, AST 24 IU/L, ALT 33 IU/L, CRP 9.16 mg/dL, and erythrocyte sedimentation rate 86 mm/h. Echocardiograms revealed pericardial effusion without coronary arterial dilation (right coronary artery (RCA) 2.5 mm, Z-score 1.37; left main coronary trunk (LMT) 2.7 mm, Z-score 1.04; left anterior descending artery (LAD) 2.4 mm, Z-score 1.39; and left circumflex artery (LCX) 2.4 mm, Z-score 1.82). These changes were also consistent with KD, and we, therefore, diagnosed the patient with KD.

At the diagnosis of KD, the patient's Kobayashi et al. risk score [9] was 6 points, which indicated the positive predictive value of no response to initial intravenous immunoglobulin (IVIG). Therefore, the patient was treated with IVIG (2 g/kg), intravenous prednisolone (PSL) (2 mg/kg/day), and oral aspirin (30 mg/kg/day) according to the strategy of the RAISE study, a multicenter, prospective, randomized trial in Japan [10]. Rapid improvement of the multiple inflammatory manifestations was observed the next day. The cellulitis also improved gradually, and she had lamellar desquamation of the left inguinal region on day 11 (Figure 1(c)). Her pericardial effusion resolved with no coronary arterial dilation (RCA 2.1 mm, Z-score 0.26; LMT 2.7 mm, Z-score 1.04; LAD 2.1 mm, Z-score 0.57; and LCX 1.5 mm, Z-score −0.60), and she was discharged on day 16. PSL was administered at 2 mg/kg/day for 12 days and, then, tapered to 1 mg/kg/day for 5 days, to 0.5 mg/kg/day for 5 days, and then, discontinued. Aspirin was administered at 30 mg/kg/day for 3 days and, then, maintained at 5 mg/kg/day until 2 months after the disease onset. Her symptoms did not relapse after discharge despite the tapering and discontinuation of the PSL and aspirin. Two months after discharge, the local findings of the cellulitis were completely resolved. Echocardiograms remained normal for 1 year after discharge (RCA 2.4 mm, Z-score 0.75; LMT 2.8 mm, Z-score 0.96; LAD 1.9 mm, Z-score -0.32; and LCX 1.8 mm, Z-score 0.02 at 1-year follow-up).

**Figure 4:**
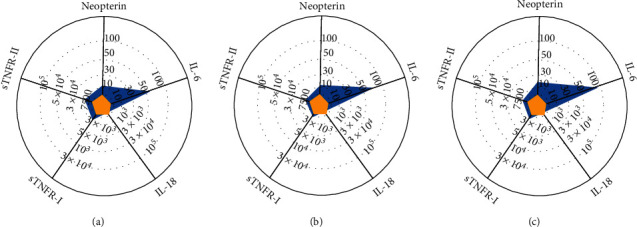
Serum cytokine profiles. (a) At admission. (b) Before intravenous immunoglobulin treatment. (c) Typical KD patients (*N* = 64). Overlaid inner yellow pentagons in the chart of KD patients show the mean values of healthy controls. IL, Interleukin; KD, Kawasaki disease; sTNFR, soluble tumor necrosis factor receptor.

To investigate the pathophysiology of this case, we sequentially measured serum cytokine levels from admission to initiation of the treatment with IVIG and PSL. Serum levels of neopterin (14.8, normal < 5 nmol/L), interleukin (IL)-6 (56, normal < 3 pg/mL), soluble tumor necrosis factor receptor type I (sTNFR-I) (2720, normal 484–1407 pg/mL), and sTNFR-II (7580, normal 829–2262 pg/mL) were elevated, whereas serum levels of IL-18 (315, normal < 500 pg/mL) were normal. This pattern is consistent with that of KD (Figure 4) and was sustained until the initiation of IVIG and PSL (Figures 2 and 4).

## 3. Discussion

Several reports in the current literature describe KD as initially preceded by cellulitis [3–7, 11–14]. However, an initial presentation with inguinal cellulitis seems to be extremely rare [7], and, to our knowledge, no cases have reported a cytokine profile analysis in KD patients with cellulitis.

Cellulitis is a frequently encountered bacterial inflammation of the deep dermis and subcutaneous tissue, with redness, pain, and swelling [15]. Any area, such as the ears, trunk, fingers, and toes, can be affected [16]; however, inguinal cellulitis is not common. Therefore, in this case, many differential diagnoses, such as erysipelas, necrotizing fasciitis, septic arthritis, and deep vein thrombosis (DVT), were considered carefully [17]. Erysipelas is a more superficial infection than cellulitis, affecting the upper dermis and superficial lymphatic system [17]. It is mainly caused by group A *Streptococcus pyogenes*, and the first-line treatment for erysipelas is an antibiotic such as amoxicillin or cephalexin [17]. In this case, the antigen testing of group A streptococcus was negative and there was no response to antibiotic treatment. CT images also showed that the infection was not limited to the superficial area; thus, erysipelas was not likely. Necrotizing fasciitis is a rare life-threatening infection of the fascia that can lead to rapid local tissue destruction, necrosis, and severe sepsis [18]. The laboratory risk indicator for the necrotizing fasciitis score [19], in which a score of 6 or greater confers a higher risk of necrotizing fasciitis, on the day of admission was 3 in this case. In addition, CT imaging revealed no subcutaneous gas or necrosis in the inguinal soft tissue, indicating necrotizing fasciitis was less likely. Septic arthritis is a medical emergency which commonly exhibits monoarticular joint pain with erythema, warmth, and swelling [20]. It can involve any joint but typically involves the knee joint [17]. The patient had no involvement in her knee joint, but her inguinal erythema expanded near her hip joint. However, she did not present with hip joint pain or decreased mobility of the hip joint, implying less possibility of septic arthritis. DVT typically presents with tenderness, erythema, warmth, and edema, often affecting the lower extremities [17]. Patients typically have risk factors for DVT such as an unmovable state, active cancer, or a family history of venous thromboembolism [17]. The current patient had no risk factors for DVT, and her CT imaging findings did not detect DVT. Consequently, her diagnosis of cellulitis had become reliable.

The KD patient with inguinal cellulitis reported by Itamura et al. [7] initially presented with right inguinal pain. Erythema, tenderness, and swelling were, then, observed. The patient did not respond to antibiotics and, subsequently, had symptoms of KD [7]. The clinical course was similar to our case. In addition, several previous reports of KD with cellulitis also indicated no infectious etiology, poor response to antibiotics, and rapid improvement immediately following the administration of IVIG and/or other anti-inflammatory agents [3, 11–13]. From the lack of infectious etiology and poor response to antibiotics, it is speculated that the cellulitis was not a simple bacterial infection, but rather an invasion of activated inflammatory cells, as well as KD.

The etiology of KD is unknown, and no specific diagnostic markers have been reported. The diagnosis of KD is, therefore, based on clinical signs. Atypical initial presentations, such as cellulitis and absence of subsequent typical signs, make rapid and accurate diagnosis difficult and may delay treatment, which may cause coronary sequelae. Çerman et al. [14] reported a case of incomplete KD preceded by orbital cellulitis and pansinusitis, with subsequent coronary aneurysm formation. The authors concluded that when cellulitis with a poor response to antibiotics is observed, attention should be paid to the possibility of underlying KD and the risk of subsequent coronary arterial abnormalities, even if few other signs of KD are present. Fortunately, in our case, typical signs of KD emerged in the early phase, making the diagnosis relatively straightforward. As a result, there was no treatment delay or sequelae. However, early diagnosis is difficult in atypical cases, such as that reported by Çerman et al. [14]. Therefore, when cellulitis refractory to antibiotics is observed, KD (including complete and incomplete types) should be added to the differential diagnosis at an early stage. In addition, echocardiograms should be performed frequently enough to prevent coronary sequelae formation from delayed treatment.

To investigate the pathophysiology of the cellulitis initially observed in this case, we sequentially measured serum cytokine levels from admission to the initiation of IVIG and PSL. Previous reports showed that serum cytokine profile analysis is useful for distinguishing KD from other diseases and that despite having no reliable reference value as a quantitative criterion of IL-6, IL-6 is a key cytokine in the pathogenesis of KD [21–23]. Interestingly, this patient's serum cytokine profile at admission was consistent with the IL-6 dominant pattern shown in KD patients, despite her initial lack of KD symptoms. Furthermore, this IL-6 dominant pattern was sustained until the initiation of IVIG and PSL. The elevation of serum IL-6 level also occurs in patients with bacterial infection [23, 24]. Thus, bacterial infection could not be ruled out. However, the profile pattern found at admission and before the initiation of IVIG and PSL in this case was extremely similar to that of KD, suggesting that the initial inguinal inflammation might have been associated with KD, and perhaps, this was not a case of secondary KD following advanced ordinal cellulitis. However, since this is a case report, our findings cannot be generalized. Further studies are required to determine whether this can be applied to all cases of KD with atypical manifestations.

## 4. Conclusions

We reported a case of KD with an initial manifestation mimicking bacterial inguinal cellulitis. KD should be included in the differential diagnosis for patients exhibiting a manifestation of inguinal cellulitis and having no response to initial empiric antibiotics.

## Data Availability

The data used in this report are available from the corresponding author on reasonable request.

## Abbreviations

ALT: Alanine aminotransferase
AST: Aspartate aminotransferase
CRP: C-reactive protein
CT: Computed tomography
DVT: Deep vein thrombosis
IL: Interleukin
IVIG: Intravenous immunoglobulin
KD: Kawasaki disease
LAD: Left anterior descending artery
LCX: Left circumflex artery
LMT: Left main coronary trunk
PLT: Platelets
PSL: Prednisolone
RCA: Right coronary artery
sTNFR: Soluble tumor necrosis factor receptor
WBC: White blood cell count.
